# Unravelling the health status of brachycephalic dogs in the UK using multivariable analysis

**DOI:** 10.1038/s41598-020-73088-y

**Published:** 2020-10-14

**Authors:** D. G. O’Neill, C. Pegram, P. Crocker, D. C. Brodbelt, D. B. Church, R. M. A. Packer

**Affiliations:** 1 Pathobiology and Population Sciences, The Royal Veterinary College, Hawkshead Lane, North Mymms, Hatfield, AL9 7TA Herts UK; 2 Clinical Sciences and Services, The Royal Veterinary College, Hawkshead Lane, North Mymms, Hatfield, AL9 7TA Herts UK

**Keywords:** Evolution, Zoology, Biomarkers, Diseases, Medical research, Risk factors

## Abstract

Brachycephalic dog breeds are regularly asserted as being less healthy than non-brachycephalic breeds. Using primary-care veterinary clinical data, this study aimed to identify predispositions and protections in brachycephalic dogs and explore differing inferences between univariable and multivariable results. All disorders during 2016 were extracted from a random sample of 22,333 dogs within the VetCompass Programme from a sampling frame of 955,554 dogs under UK veterinary care in 2016. Univariable and multivariable binary logistic regression modelling explored brachycephaly as a risk factor for each of a series of common disorders. Brachycephalic dogs were younger, lighter and less likely to be neutered than mesocephalic, dolichocephalic and crossbred dogs. Brachycephalic differed to non-brachycephalic types in their odds for 10/30 (33.33%) common disorders. Of these, brachycephalic types were predisposed for eight disorders and were protected for two disorders. Univariable and multivariable analyses generated differing inference for 11/30 (30.67%) disorders. This study provides strong evidence that brachycephalic breeds are generally less healthy than their non-brachycephalic counterparts. Results from studies that report only univariable methods should be treated with extreme caution due to potential confounding effects that have not been accounted for during univariable study design or analysis.

## Introduction

Issues relating to the health and welfare concerns for brachycephalic dogs have become an increasingly high profile topic in veterinary medicine in the UK^1,2^ and internationally^3,4^ over the past decade. Controversy surrounds these much cherished but often debated breeds, with marked rises in their popularity^5^ set against a backdrop of increasing evidence of the health compromises associated with their exaggerated body morphology^6,7^. Indeed, some veterinarians now consider the health and welfare of several popular brachycephalic breeds too compromised to justify their continued breeding^8^. A growing body of evidence is accumulating to suggest that brachycephalic breeds are strongly predisposed to a range of disorders intrinsically related to their typical conformations, including respiratory disease^9,10^, eye disease^11,12^, dystocia^13,14^, spinal disease^15^, heat stroke and pneumonia^16^. Brachycephalic breeds are also reported with significantly shorter lifespans (median longevity: 8.6 years) than moderate and non-brachycephalic dogs (median 12.7 years)^17^.

To date, a priori exploration of brachycephalic health has often compared brachycephalic and non-brachycephalic dogs for individual disorders selected based on prior belief of a brachycephalic predisposition e.g. upper respiratory tract disease^9^. An alternative approach that encompasses a wider spectrum of disorders has been to report prevalence within key individual brachycephalic breeds such as French Bulldogs, Bulldogs and Pugs but without directly including a comparator group of dogs for reference^18–20^. While both approaches are useful, inference from these approaches is limited to the specific disorder or breed under study, and it is challenging to draw the deeper conclusions across the spread of disorders and breeds that are needed to fully evaluate health associations for brachycephalic dogs. To date, an analysis of pet insurance data in the United States of America is the only study to have directly compared the overall health of brachycephalic versus non-brachycephalic dogs overall across a range of common disorders simultaneously^16^. That insurance study reported higher prevalence in brachycephalic dogs for many health problems. However, these data were derived from an inherently biased subset of the overall population that were insured and formal statistical methods such as multivariable analysis to account for potential confounding variables such as differing age and neutering structures between the groups in the study population were not applied^21^.

In the UK, the brachycephalic population of dogs has some unique demographic features compared with non-brachycephalic dogs. Rapid popularity increases over the past decade for some brachycephalic breeds such as French Bulldog and Pug have led to disproportionate numbers of younger animals representing these breeds^18,19^. Age is consistently shown as one of the most important risk factors for disease; young animals show higher rates of infectious disease rates^22^ but lower rates of neoplastic (e.g. lymphoma^23^), musculoskeletal (e.g. elbow joint disease^24^) and degenerative disease (e.g. osteoarthritis^25^). As such, univariable analyses of brachycephalic health parameters are likely to be highly confounded by age and therefore it seems sensible to account for potential confounders in analyses of breed health as a general rule^21^.

Much of the previous veterinary literature on breed predispositions reported findings from univariable analyses that compared breed effects without accounting for other differences such as age^7^. It is possible this approach may lead to false positive and false negative findings^26^. Many of the more recent VetCompass studies of canine health have highlighted the complexity underlying disorder risk, with variables such as age, bodyweight, neutering and insurance status commonly associated with disease risk factors in addition to breed effects^14,24,27–29^. As such, the secondary aim of the study was to compare the consistency of findings between univariable versus multivariable analyses to assist in gaining a deeper understanding of the reliability of univariable analyses for robust inference on factors related to dog health.

The VetCompass Programme that collects anonymised data from primary-care veterinary practices was used to compare overall health between large groups of dogs^30^. VetCompass has previously been applied to report that purebred dogs had higher prevalence than crossbreds for 3/20 of the most-frequently recorded disorders, although these analyses were limited to univariable statistical methods^31^. A similarly holistic approach, but extended to include multivariable methods, could compare health between brachycephalic versus non-brachycephalic dogs to investigate whether brachycephalic dogs are predisposed or protected to common disorders after accounting for other demographic factors. The findings from such an approach would offer further insights into both the challenges, and indeed potential benefits, to health and welfare from being a brachycephalic breed. With this background, using anonymised veterinary clinical data from the VetCompass Programme^30^, the primary aim of the current study was to compare the general demography and prevalence of common disorders between brachycephalic dogs compared with mesocephalic and dolichocephalic types under primary veterinary care in the UK during 2016 and specifically to identify disorders with predisposition and protection in the brachycephalic dogs compared with non-brachycephalic dogs. A secondary aim was to explore differences in the results from univariable compared with multivariable risk factor analyses in order to better understand the value of accounting for confounding in breed health studies. These results could assist welfare scientists, breeders, kennel clubs, veterinary practitioners, owners and other stakeholder with an evidence base on the health of the wider general population of brachycephalic dogs that could assist to predict, prevent and manage key health and welfare opportunities for brachycephalic dog types.

## Results

### Demography

The study included a random sample of 22,333 dogs attending 784 veterinary clinics from an overall population of 955,554 dogs under veterinary care in 2016. The analysis included 22,248/22,333 (99.62%) dogs with breed information available. These dogs included 4,169 (18.74%) brachycephalic, 10,341 (46.48%) mesocephalic, 1,744 (7.84%) dolichocephalic and 5,994 (26.94%) crossbred types. At a more summarised level, there were 4,169 (18.74%) brachycephalic and 18,079 (81.26%) non-brachycephalic types (Table 1).

**Table 1:** Demography of brachycephalic (n = 4,169), mesocephalic (n = 10,341), dolichocephalic (n = 1,744) and crossbred (n = 5,994) dog types under UK primary veterinary care from January 1st 2016 to December 31st, 2016 at practices participating in the VetCompass Programme. *Count covers dogs with available data.

Variable	Category	Brachycephalic No, (%)*	Mesocephalic No, (%)*	Dolichocephalic No, (%)*	Crossbred No, (%)*
Sex	Female	1,982 (47.67)	4,796 (46.52)	812 (46.72)	2,905 (48.61)
	Male	2,176 (52.33)	5,513 (53.48)	926 (53.28)	3,071 (51.39)
Neutered—Female	Entire	1,267 (63.93)	2,524 (52.63)	418 (51.48)	1,440 (49.57)
	Neutered	715 (36.07)	2,272 (47.37)	394 (48.52)	1,465 (50.43)
Neutered—Male	Entire	1,365 (62.73)	3,029 (54.94)	513 (55.40)	1,551 (50.50)
	Neutered	811 (37.27)	2,484 (45.06)	413 (44.60)	1,520 (49.50)
Adult bodyweight (≥ 18 months) (kg)	< 5.0	443 (17.11)	403 (5.68)	63 (5.44)	191 (4.72)
	5.0 to < 10.0	1,154 (44.57)	1,745 (24.60)	215 (18.55)	1,188 (29.33)
	10.0 to < 15.0	511 (19.74)	1,039 (14.65)	138 (11.91)	770 (19.01)
	15.0 to < 20.0	55 (2.12)	1,034 (14.58)	61 (5.26)	538 (13.28)
	20.0 to < 25.0	73 (2.82)	935 (13.18)	83 (7.16)	511 (12.62)
	25.0 to < 30.0	119 (4.60)	697 (9.83)	170 (14.67)	360 (8.89)
	30.0 to < 35.0	92 (3.55)	587 (8.27)	179 (15.44)	213 (5.26)
	35.0 to < 40.0	47 (1.82)	378 (5.33)	126 (10.87)	145 (3.58)
	≥ 40.0	95 (3.67)	276 (3.89)	124 (10.70)	134 (3.31)
Age (years)	< 1.0	632 (15.38)	956 (9.32)	167 (9.73)	746 (12.60)
	1.0 to < 2.0	786 (19.12)	1,271 (12.39)	224 (13.05)	988 (16.69)
	2.0 to < 4.0	927 (22.55)	1,825 (17.79)	312 (18.18)	1,380 (23.31)
	4.0 to < 6.0	661 (16.08)	1,600 (15.60)	275 (16.03)	908 (15.34)
	6.0 to < 8.0	495 (12.04)	1,412 (13.77)	251 (14.63)	634 (10.71)
	8.0 to < 10.0	301 (7.32)	1,219 (11.88)	228 (13.29)	494 (8.34)
	10.0 to < 12.0	171 (4.16)	940 (9.16)	139 (8.10)	321 (5.42)
	≥ 12.0	137 (3.33)	1,034 (10.08)	120 (6.99)	450 (7.60)
Insurance	Non-insured	3,691 (88.53)	8,875 (85.82)	1,464 (83.94)	5,242 (87.45)
	Insured	478 (11.47)	1,466 (14.18)	280 (16.06)	752 (12.55)

The median age of brachycephalic dog types was 3.31 years (IQR 1.40–6.24, range 0.15–19.54). Brachycephalic dog types were younger than mesocephalic types (median 5.33 years, IQR 2.33–8.98, range 0.01–20.46) (*P* < 0.001), dolichocephalic types (5.07 years, IQR 2.23–8.49, range 0.20–18.33) (*P* < 0.001) and crossbred types (3.74 years, IQR 1.68–7.34, range 0.01–19.49) (*P* < 0.001). The median adult bodyweight of brachycephalic dog types was 8.75 kg (IQR 6.29–12.18, range 1.52–80.63). Brachycephalic dog types were lighter than mesocephalic types (median 16.98 kg, IQR 8.93–25.95, range 1.41–83.70) (*P* < 0.001), dolichocephalic types (25.80 kg, IQR 10.30–33.78, range 2.20–76.77) (*P* < 0.001) and crossbred types (13.80 kg, IQR 8.43–23.34, range 2.10–85.00) (*P* < 0.001). There was no overall association between sex and the skull conformation group (*P* = 0.069). Pairwise proportional neutering was lower in brachycephalic types (number neutered 1,526, 36.70%) than mesocephalic types (4,756, 46.13%) (*P* < 0.001), dolichocephalic types (807, 46.43%) (*P* < 0.001) and crossbred types (2,985, 49.95%) (*P* < 0.001). Pairwise proportional insurance did not differ between brachycephalic (478, 11.47%) and crossbreds (752, 12.55%) (*P* = 0.101) but insurance was lower for brachycephalic types than for mesocephalic (1,466, 14.18%) (*P* < 0.001) or dolichocephalic types (280, 16.06%) *P* < 0.001) (Table 2).

**Table 2:** Breed composition for the 15 most common breeds of brachycephalic (n = 4,169), mesocephalic (n = 10,341), dolichocephalic (n = 1,744) and crossbred (n = 5,994) dog types under UK primary veterinary care from January 1st 2016 to December 31st, 2016 at practices participating in the VetCompass Programme.

Rank	Brachycephalic breed	No. (%)	Mesocephalic breed	No. (%)	Dolichocephalic breed	No. (%)	Crossbreeds	No. (%)
1	Chihuahua	955 (22.91)	Labrador Retriever	1462 (14.14)	German Shepherd Dog	546 (31.31)	Crossbreed	4699 (78.4)
2	Shih-tzu	795 (19.07)	Staffordshire Bull Terrier	1304 (12.61)	Greyhound	149 (8.54)	Cockapoo	478 (7.97)
3	Cavalier King Charles Spaniel	435 (10.43)	Jack Russell Terrier	1190 (11.51)	Whippet	117 (6.71)	Labradoodle	175 (2.92)
4	Pug	413 (9.91)	Cocker Spaniel	771 (7.46)	Miniature Dachshund	111 (6.36)	Lurcher	135 (2.25)
5	French Bulldog	398 (9.55)	Yorkshire Terrier	765 (7.4)	Toy Poodle	77 (4.42)	Cavapoo	104 (1.74)
6	Lhasa Apso	311 (7.46)	Border Collie	608 (5.88)	Standard Doberman Pinscher	65 (3.73)	Sprocker	89 (1.48)
7	Boxer	245 (5.88)	West Highland White Terrier	516 (4.99)	English Bull Terrier	64 (3.67)	Cavachon	73 (1.22)
8	British Bulldog	209 (5.01)	English Springer Spaniel	475 (4.59)	Basset Hound	59 (3.38)	Jug	40 (0.67)
9	American Bulldog	81 (1.94)	Bichon Frise	336 (3.25)	Miniature Poodle	54 (3.1)	Puggle	38 (0.63)
10	King Charles Spaniel	73 (1.75)	Border Terrier	258 (2.49)	Dachshund	47 (2.69)	Goldendoodle	29 (0.48)
11	Dogue de Bordeaux	72 (1.73)	Golden Retriever	241 (2.33)	Scottish Terrier	34 (1.95)	Jackapoo	29 (0.48)
12	Bull Mastiff	48 (1.15)	Miniature Schnauzer	217 (2.1)	Scottish Rough Collie	30 (1.72)	Chorkie	19 (0.32)
13	Boston Terrier	35 (0.84)	Beagle	197 (1.91)	Poodle	27 (1.55)	Shih-Poo	13 (0.22)
14	Olde English Bulldogge	19 (0.46)	Rottweiler	175 (1.69)	Lakeland Terrier	26 (1.49)	Schnoodle	12 (0.2)
15	Pekingese	12 (0.29)	Pomeranian	139 (1.34)	Standard Poodle	26 (1.49)	Malti-Poo	10 (0.17)
	Other	68 (1.63)	Other	1687 (16.31)	Other	312 (17.89)	Other	51 (0.85)

The brachycephalic group included 34 individual breeds, the mesocephalic group included 169 individual breeds and the dolichocephalic group included 66 individual breeds (Supplementary A). The most common brachycephalic breeds were Chihuahua (n = 955, 22.91%), Shih-tzu (795, 19.07%) and Cavalier King Charles Spaniel (435, 10.43%). The most common mesocephalic breeds were Labrador Retriever (1462, 14.14%), Staffordshire Bull Terrier (1304, 12.61%) and Jack Russell Terrier (1190, 11.51%). The most common dolichocephalic breeds were German Shepherd Dog (546, 31.31%), Greyhound (149, 8.54%) and Whippet (117, 6.71%). The most common crossbred types were non-designer type crossbreds (4699, 78.4%), Cockapoo (478, 7.97%) and Labradoodle (175, 2.92%). The 15 most common breeds within each skull shape group comprised a greater proportion of the brachycephalic dog types (4,101/4,169, 98.37%) than mesocephalic types (8,654/10,341, 83.69%) (*P* < 0.001) or dolichocephalic types (1,432/1,744, 82.11%) (*P* < 0.001) (Table 2).

### Precise diagnoses

In the overall study sample, there were 14,704 (65.84%) dogs with at least one disorder during the 12-month interval from January to December 2016. The proportion of dogs with at least one disorder recorded during 2016 for the skull shape conformation were: brachycephalic dog types n = 2,769 (66.42%), mesocephalic types n = 6,868 (66.42%), dolichocephalic types n = 1,160 (66.51%) and crossbred types n = 3,866 (64.50%). Univariable logistic regression modelling showed that brachycephalic (odds ratio [OR] 1.09 (95% confidence interval [CI] 1.00–1.18, *P* = 0.045) and mesocephalic (OR 1.09, 95% CI 1.02–1.16, *P* = 0.013) types had higher odds of having at least one disorder compared with crossbred types, whereas no difference was identified for dolichocephalic types (OR 1.09, 95% CI 00.98–1.22, *P* = 0.120). Multivariable logistic regression modelling (adjusting for adult bodyweight, bodyweight relative to breed/sex mean, age, sex, neuter and insurance) revealed an increased odds of having at least one disorder in brachycephalic types (OR 1.27, 95% confidence interval [CI] 1.13–1.43, *P* < 0.001) compared with crossbred types. However, mesocephalic types (OR 0.95, 95% CI 0.87–1.04, *P* = 0.285) and dolichocephalic types (OR 0.96, 95% CI 0.83–1.12, *P* = 0.625) were not significantly different to crossbreds. The 95% CI for mesocephalic and dolichocephalic dogs did not overlap the 95% CI for brachycephalic dogs, indicating that brachycephalic dogs also had increased odds of having at least one disorder compared with mesocephalic and dolichocephalic dogs.

The median count of disorders in the overall study sample during 2016 was 1 (interquartile range [IQR] 0–2, range 0–17). Univariable Poisson regression modelling showed that brachycephalic (disorder count risk ratio [DCRR] 1.13, 95% CI 1.10–1.17, *P* < 0.001), mesocephalic (DCRR 1.13, 95% CI 1.10–1.16, *P* < 0.001) and dolichocephalic types (DCRR 1.11. 95% CI 1.06–1.16, *P* < 0.001) had higher disorder count risk ratios than crossbred types. Multivariable Poisson regression modelling (adjusting for adult bodyweight, bodyweight relative to breed/sex mean, age, sex, neuter and insurance) showed an increased disorder count risk ratio for brachycephalic types (DCRR 1.24, 95% CI 1.19–1.29, *P* < 0.001) and a decreased disorder count risk ratio for mesocephalic types (DCRR 1.04, 95% CI 1.01–1.08, *P* = 0.007) compared to crossbreds. Dolichocephalic types (DCRR 1.04. 95% CI 0.99–1.10, *P* = 0.124) were no longer significantly different to crossbreds.

The most common precise disorders (i.e. greatest prevalence) in the brachycephalic types were periodontal disease (n = 485, prevalence = 11.63%), otitis externa (303, 7.27%), obesity (266, 6.38%), anal sac impaction (249, 5.97%), overgrown nail(s) (212, 5.09%), diarrhoea (143, 3.43%) and heart murmur (3.43%). There were eight precise disorders with the higher odds in multivariable logistic regression analysis (i.e. disorder predisposition) for brachycephalic types compared with non-brachycephalic types: corneal ulceration (OR 8.40, 95% confidence interval [CI] 5.21–13.56, *P* < 0.001), heart murmur (OR 3.52, 95% CI 2.70–4.60, *P* < 0.001), umbilical hernia (OR 3.16, 95% CI 1.94–5.18, *P* < 0.001), pododermatitis (OR 1.66, 95% CI 1.20–2.28, *P* = 0.002), skin cyst (OR 1.52, 95% CI 1.04–2.22, *P* = 0.029), patellar luxation (OR 1.40, 95% CI 1.02–1.93, *P* = 0.038), otitis externa (OR 1.29, 95% CI 1.10–1.51, *P* = 0.002) and anal sac impaction (OR 1.24, 95% CI 1.03–1.50, *P* = 0.021). Two precise disorders had reduced odds for brachycephalic types in multivariable analysis: undesirable behaviour (OR 0.52, 95% CI 0.34–0.81, *P* = 0.003) and claw injury (OR 0.45, 95% CI 0.29—0.70, *P* < 0.001) (Table 3).

**Table 3:** Prevalence of the thirty most common *precise* disorders recorded in brachycephalic (n = 4,169) and non-brachycephalic (n = 18,079) dog types under UK primary veterinary care from January 1st 2016 to December 31st, 2016 at practices participating in the VetCompass Programme.

Precise disorder term	Brachycephalic No. (%)	Non-brachycephalic No. (%)	Univariable logistic regression			Multivariable logistic regression		
			OR^1^	95% CI^2^	*P *value^3^	OR	95% CI	*P *value*
Corneal ulceration	100 (2.40)	72 (0.40)	6.15	4.53–8.34	** < 0.001**	8.40	5.21–13.56	** < 0.001**
Heart murmur	143 (3.43)	330 (1.83)	1.91	1.57–2.33	** < 0.001**	3.52	2.70–4.60	** < 0.001**
Umbilical hernia	91 (2.18)	117 (0.65)	3.43	2.60–4.51	** < 0.001**	3.16	1.94–5.18	** < 0.001**
Pododermatitis	71 (1.70)	230 (1.27)	1.35	1.03–1.76	**0.031**	1.66	1.20–2.28	**0.002**
Skin cyst*	50 (1.20)	196 (1.08)	1.11	0.81–1.51	0.522	1.52	1.04–2.22	**0.029**
Patellar luxation	86 (2.06)	146 (0.81)	2.59	1.98–3.38	** < 0.001**	1.40	1.01–1.93	**0.038**
Otitis externa*	303 (7.27)	1323 (7.32)	0.99	0.87–1.13	0.911	1.29	1.10–1.51	**0.002**
Retained deciduous tooth*	88 (2.11)	137 (0.76)	2.82	2.16–3.70	** < 0.001**	1.30	0.85–2.01	0.221
Pyoderma	67 (1.61)	258 (1.43)	1.13	0.86–1.48	0.383	1.26	0.92–1.74	0.156
Anal sac impaction	249 (5.97)	822 (4.55)	1.33	1.15–1.54	** < 0.001**	1.24	1.03–1.50	**0.021**
Pruritus	81 (1.94)	282 (1.56)	1.25	0.97–1.61	0.079	1.22	0.90–1.67	0.203
Overgrown nail(s)*	212 (5.09)	760 (4.20)	1.22	1.05–1.43	**0.012**	1.18	0.98–1.43	0.102
Wound	42 (1.01)	208 (1.15)	0.87	0.63–1.22	0.430	1.15	0.77–1.72	0.497
Disorder not diagnosed*	20 (0.48)	161 (0.89)	0.54	0.34–0.86	**0.009**	1.09	0.55–2.16	0.805
Allergy	66 (1.58)	284 (1.57)	1.01	0.77–1.32	0.954	1.06	0.76–1.48	0.709
Diarrhoea	143 (3.43)	706 (3.91)	0.87	0.73–1.05	0.149	1.05	0.82–1.33	0.710
Gastroenteritis	64 (1.54)	233 (1.29)	1.19	0.90–1.58	0.212	1.05	0.74–1.51	0.778
Skin mass*	57 (1.37)	406 (2.25)	0.60	0.46–0.80	** < 0.001**	1.01	0.73–1.39	0.972
Lameness*	88 (2.11)	502 (2.78)	0.76	0.60–0.95	**0.016**	0.99	0.74–1.31	0.922
Flea infestation	101 (2.42)	356 (1.97)	1.23	0.99–1.55	0.063	0.98	0.73–1.31	0.878
Obesity*	266 (6.38)	1311 (7.25)	0.87	0.76–1.00	**0.048**	0.96	0.81–1.14	0.657
Vomiting	131 (3.14)	546 (3.02)	1.04	0.86–1.26	0.679	0.96	0.74–1.24	0.748
Periodontal disease*	485 (11.63)	2310 (12.78)	0.90	0.81–1.00	**0.045**	0.93	0.81–1.07	0.308
Aggression	86 (2.06)	414 (2.29)	0.90	0.71–1.14	0.373	0.91	0.67–1.22	0.511
Conjunctivitis	86 (2.06)	413 (2.28)	0.90	0.71–1.14	0.384	0.89	0.65–1.22	0.464
Foreign body	40 (0.96)	241 (1.33)	0.72	0.51–1.00	0.053	0.80	0.52–1.24	0.323
Osteoarthritis*	39 (0.94)	483 (2.67)	0.34	0.25–0.48	** < 0.001**	0.79	0.53–1.16	0.230
Lipoma*	17 (0.41)	303 (1.68)	0.24	0.15–0.39	** < 0.001**	0.59	0.34–1.01	0.056
Undesirable behaviour	42 (1.01)	291 (1.61)	0.62	0.45–0.86	**0.004**	0.52	0.34–0.81	**0.003**
Claw injury	31 (0.74)	278 (1.54)	0.48	0.33–0.70	** < 0.001**	0.45	0.29–0.70	** < 0.001**

The probability for each disorder in brachycephalic compared with non-brachycephalic dogs is reported using univariable (binary logistic regression) and multivariable methods (mixed effects multivariable logistic regression modelling that included the *skull conformation*, *adult bodyweight category, bodyweight relative to breed/sex mean, age category, sex, neuter* and *insurance* with the *clinic* attended included as a random effect). *P *values < 0.05 are shown in bold.

*Differing inference between univariable and multivariable results. ^1^OR odds ratio. ^2^CI confidence interval. ^3^*P *value comparing brachycephalic versus non-brachycephalic for each disorder.

**Table 4:** Prevalence of the sixteen most common *grouped* disorders recorded in brachycephalic (n = 4,169) and non-brachycephalic (n = 18,079) dog types under UK primary veterinary care from January 1st 2016 to December 31st, 2016 at practices participating in the VetCompass Programme.

Grouped disorder term	Brachycephalic No. (%)	Non-brachycephalic No. (%)	Univariable logistic regression			Multivariable logistic regression		
			OR^1^	95% CI^2^	*P *value^3^	OR	95% CI	*P *value*
Cardiac	199 (4.77)	431 (2.38)	2.05	1.73–2.44	** < 0.001**	4.06	3.18–5.18	** < 0.001**
Ophthalmological	393 (9.43)	1168 (6.46)	1.51	1.34–1.70	** < 0.001**	1.80	1.53–2.11	** < 0.001**
Upper respiratory tract	208 (4.99)	576 (3.19)	1.60	1.36–1.88	** < 0.001**	1.62	1.30–2.03	** < 0.001**
Aural*	342 (8.20)	1478 (8.18)	1.00	0.89–1.14	0.952	1.33	1.14–1.55	** < 0.001**
Dermatological	587 (14.08)	2217 (12.26)	1.17	1.06–1.29	**0.001**	1.32	1.16–1.50	** < 0.001**
Anal sac	300 (7.20)	947 (5.24)	1.40	1.23–1.61	** < 0.001**	1.33	1.12–1.58	**0.001**
Mass/swelling*	152 (3.65)	1013 (5.60)	0.64	0.54–0.76	** < 0.001**	1.20	0.97–1.47	0.088
Neoplasia*	145 (3.48)	992 (5.49)	0.62	0.52–0.74	** < 0.001**	1.12	0.90–1.38	0.313
Musculoskeletal*	296 (7.10)	1630 (9.02)	0.77	0.68–0.88	** < 0.001**	1.11	0.94–1.31	0.207
Parasitic	175 (4.20)	671 (3.71)	1.14	0.96–1.35	0.139	1.07	0.85–1.34	0.557
Trauma	132 (3.17)	687 (3.80)	0.83	0.69–1.00	0.050	1.01	0.79–1.29	0.920
Enteropathy	424 (10.17)	1900 (10.51)	0.96	0.86–1.08	0.519	1.00	0.86–1.16	0.992
Claw/nail	307 (7.36)	1269 (7.02)	1.05	0.93–1.20	0.434	1.00	0.85–1.18	0.998
Obesity*	266 (6.38)	1311 (7.25)	0.87	0.76–1.00	**0.048**	0.96	0.81–1.14	0.657
Dental	582 (13.96)	2563 (14.18)	0.98	0.89–1.08	0.718	0.94	0.82–1.07	0.345
Behavioural	169 (4.05)	967 (5.35)	0.75	0.63–0.88	**0.001**	0.73	0.59–0.90	**0.004**

The probability for each disorder in brachycephalic compared with non-brachycephalic dogs is reported using univariable (binary logistic regression) and multivariable methods (mixed effects multivariable logistic regression modelling that included the *skull conformation*, *adult bodyweight category, bodyweight relative to breed/sex mean, age category, sex, neuter* and *insurance* with the *clinic* attended included as a random effect). *P *values < 0.05 are shown in bold.

* Differing inference between univariable and multivariable results. ^1^OR odds ratio. ^2^CI confidence interval. ^3^*P *value comparing brachycephalic versus non-brachycephalic for each disorder.

Based on the multivariable logistic regression analysis results for the individual precise disorders, the odds of ten of the thirty (10/30; 33.33%) disorders differed between brachycephalic and non-brachycephalic types, with 8/10 disorders showing higher odds in brachycephalic types while 2/10 disorders had lower odds in brachycephalic types. Review of the univariable logistic regression analyses identified 17/30 (56.67%) precise disorders with differing odds between brachycephalic and non-brachycephalic types, with 8/17 disorders showing higher odds in brachycephalic types while 9/17 disorders had lower odds in brachycephalic types. Univariable and multivariable analyses generated differing inference on disorder predisposition between brachycephalic and non-brachycephalic types for 11/30 (30.67%) common precise disorders (Table 3).

### Grouped diagnoses

The most common grouped disorders in the brachycephalic types were dermatological (n = 587, prevalence = 14.08%), dental (583, 13.96%), enteropathy (424, 10.17%), ophthalmological (393, 9.43%) and aural (342, 8.2%). There were six grouped disorders with the higher odds in multivariable logistic regression analysis for brachycephalic types compared with non-brachycephalic types: cardiac (OR 4.06, 95% CI, 3.18–5.18, *P* < 0.001), ophthalmologic (OR 1.80, 95% CI 1.53–2.11, *P* < 0.001), upper respiratory tract (OR 1.62, 95% CI 1.30–2.03, *P* < 0.001), aural (OR 1.33, 95% CI 1.14–1.55, *P* < 0.001), dermatologic (OR 1.32, 95% CI 1.16–1.50, *P* < 0.001) and anal sac (OR 1.33, 95% CI 1.12–1.58, *P* = 0.001). One grouped disorder had reduced odds for brachycephalic types in multivariable analysis: behavioural (OR 0.73, 95% CI 0.59—0.90, *P* = 0.004). (Table 4).

Based on the multivariable logistic regression analysis results for the individual grouped disorders, the odds of seven of the sixteen (7/16; 43.75%) disorders differed between brachycephalic and non-brachycephalic types, with 6/7 disorders showing higher odds in brachycephalic types while 1/7 disorders had lower odds in brachycephalic types. Review of the univariable logistic regression analyses identified 10/16 (62.50%) grouped disorders with differing odds between brachycephalic and non-brachycephalic types, with 5/10 disorders showing higher odds in brachycephalic types and 5/10 disorders with lower odds in brachycephalic types. Univariable and multivariable analyses generated differing inference on disorder predisposition between brachycephalic and non-brachycephalic types for 5/16 (31.25%) common grouped disorders (Table 4).

## Discussion

This study is the first large-scale direct comparison of the health of brachycephalic versus non-brachycephalic dogs using veterinary clinical records. The results provide strong evidence to support the position that brachycephalic dogs have reduced health overall compared with non-brachycephalic dogs based on the current evaluation of the most common conditions observed in dogs attending primary care practices. Brachycephalic dogs had higher odds of having at least one disorder diagnosed compared with mesocephalic, dolichocephalic or crossbred dogs. Among the thirty individual precise disorders, brachycephalic types showed predispositions for 8/30 disorders compared with protections for just 2/30 disorders. At the more general grouped level of disorders, brachycephalic types showed predispositions in 6/16 disorders compared with protection in just 1/16 disorder. This study focused on common problems because these contribute substantially to the overall disease burden and therefore should be considered as priority issues for these breeds^32^. The power of the current study for reliable inference on the relative health status of brachycephalic dogs is strengthened by the relatively large sample size, the breadth of clinical disorders included, access to diagnoses recorded directly by veterinary professionals and the availability of health information on non-brachycephalic dogs for comparison^33^. The majority of previous studies on breed health tended to focus primarily on identification of disorder predispositions but the current study expanded this approach by also aiming to identify disorder protections as a relatively new concept in companion animal epidemiology^7^.

As well as reporting disorder predispositions, the current study also explored disorder occurrence across the types of skull shape at a more general level by comparing the counts of disorders recorded annually in the dogs of each group. Multivariable Poisson regression modelling showed that brachycephalic types had the highest disorder count risk ratio of the four skull shape groups assessed (DCRR 1.24 compared with crossbreds). Although disorder count as a welfare metric does not consider the contribution of disorder severity and duration to the overall welfare impact^32,34^, the elevated risk for overall disorder occurrence shown in the current study provides additional support that brachycephalic types in general have reduced health compared to other types.

It is noteworthy that rising popularity and ownership over the past decade of the Pug^20^, French Bulldog^18^ and Bulldog^19^ in particular has been at the heart of the growing concerns about brachycephalic health issues in dogs^35,36^. This phenomenon may give the impression that these breeds dominate the brachycephalic dog population overall. However, in contrast, the current study shows that the most common brachycephalic breeds in 2016 in the UK were the Chihuahua, Shih-tzu and Cavalier King Charles Spaniel, whereas the Pug, French Bulldog and Bulldog were just the fourth, fifth and eight most common breeds respectively. This apparent paradox may be explained by rising popularity being reflected by increasing numbers of very young dogs being added to the overall population but that it can take a decade for these new additions to overtake the counts of pre-exiting popular brachycephalic breeds.

Although the current results reflect the relative risk for brachycephalic types overall, application of the findings for health reforms based on the individual disorders will require deeper understanding of pathogenetic pathways that lead to these disorders. Vulnerability in the brachycephalic group of dogs to disease risk can be directly related to the brachycephalic skull conformation itself or may instead be associated with alternative factors such as other conformational features typically linked with brachycephaly, specific predisposition in some common brachycephalic breeds or even lifestyle differences between brachycephalic and other dog types. Several of the disorders identified with predisposition in brachycephalic dogs in the current study have causative links associated with the brachycephalic skull conformation. These disorders include upper respiratory tract disorders^10^ and corneal ulcers^11^. However, there are many others where the underlying pathophysiological pathway may not be directly linked with brachycephalism itself but happen to be very common in some brachycephalic breeds, for example heart murmurs in Cavalier King Charles Spaniels^37^, or where no clear rationale for the increased risk in brachycephalic types is clear, such as umbilical hernia, otitis externa or anal sac impaction. As such, decision-making on the most effective potential solutions and strategies to reduce the prevalence of the predisposed disorders identified here will likely differ by breed. Promotion of widespread change to average breed conformations may reduce the frequency or severity of those disorders inherently linked to specific morphological features, for example, an increased muzzle length to move a breed away from the exaggerations of the brachycephalic category would likely reduce the risk or severity of BOAS in French Bulldogs^10, 38^. In contrast, changing skull shape may offer little direct improvement to the prevalence of other predisposed disorders such as patellar luxation which appears to be associated with miniaturization of breeds (especially those < 10 kg)^39^. Miniaturisation and association with patellar luxation has been a feature for several breeds included in this study including Chihuahua (brachycephalic) as well as the Pomeranian and Yorkshire Terrier (mesocephalic)^40^.

The degree (or severity) of brachycephaly varies between breeds (a bulldog may be considered as more severely brachycephalic than a Cavalier King Charles Spaniel) but there can also be considerable variation in brachycephaly within breeds^41^. Shifting the median severity of brachycephaly towards a longer skull shape within breeds has been suggested as one option to reduce the prevalence of disorders directly linked to brachycephaly while still retaining these breeds within the overall dog population^10,11,42^. Detrimental effects from skull shape on health may be affected by other modifiable variables (risk factors) which may vary between populations of brachycephalic dogs and over time. These differences also offer opportunities to reduce the negative welfare impact of brachycephalism if carefully managed. For example, obesity has been identified as a risk factor for Brachycephalic Obstructive Airway Syndrome (BOAS)^10,43^, with bodyweight control likely to be, in part, dependent on provision of appropriate diet and exercise by individual dog owners^44^. Although the current study did not identify higher odds of obesity in brachycephalic dogs overall, the results did highlight obesity as the third most common disorder in brachycephalic dogs which suggests there is ample scope to reduce obesity in these breeds and therefore to positively impact on the respiratory compromise shown by many of these dogs. Certain popular brachycephalic breeds, such as Pugs, have also previously been reported to be at particularly high risk of obesity^20^, suggesting that effective bodyweight control may be of additional benefit to the health of specific brachycephalic breeds.

The breed-related normalisation phenomenon describes a cognitive bias whereby humans readily accept certain clinical attributes that are typical for the breeds as falling within the domain of ‘good health’ within these breeds whereas these same clinical attributes would not be accepted as consistent with ‘good health’ in dogs in general^45,46^. The belief that clinical conditions that are overwhelmingly common in certain breeds must, de facto, also be normal and therefore acceptable has been suggested to explain the reduced frequency for presentation of dogs affected with these breed-typical conditions for veterinary care^47^. Studies in the UK indicate that over half of dogs with BOAS are not presented for veterinary investigation of this disorder because their owners perceive these clinical signs (e.g. increased respiratory noise) as ‘normal for the breed’^38,48^. The common current perception by owners and veterinary professionals alike that common breed-related traits such as snoring/snorting, drooling and exercise intolerance are somehow normal and therefore consistent with health in certain breeds could be considered as a modifiable risk factor with the potential to improve welfare in brachycephalic breeds. In humans, health is defined as a ‘state of complete physical, mental and social well-being and not merely the absence of disease or infirmity’^49^. Breed and kennel clubs, the veterinary profession and welfare bodies, as key opinion leaders, should emphasise that attributes inconsistent with good health in dogs overall (for example, noisy laboured breathing at rest) should not be acceptable as consistent with good health in individual breeds; and that any exceptions to this rule should be considered as a deviation from good health.

Normalisation of expectations of health can lead to other phenomena that promote diagnostic biases between breeds. Veterinarians in clinical practice often tend to rely on intuitive methods such as pattern recognition for speedy diagnosis-making rather than following a more labour-intensive process of problem-based inductive clinical reasoning^50^. *Script theory* proposes a rationale for how clinicians store sets of pre-compiled knowledge called ‘illness scripts’ as mental models of real-world disorders; these illness scripts then influence the probability of certain diagnoses being reached^51^. Since illness scripts depend heavily on prior knowledge and beliefs, it stands to reason that increasing awareness of heightened risk of certain disorders such as corneal ulceration^12^ or dystocia^13,14^ in brachycephalic breeds will bias the probability of such diagnoses in these breeds.

The current study highlights that the 2016 UK population of brachycephalic dogs were demographically different to their mesocephalic, dolichocephalic and crossbreed counterparts in many characteristics that may be associated with health outcomes. Brachycephalic dogs were generally younger and lighter than the other three groups. The probability of the occurrence of many disorders in dogs is strongly associated with age and bodyweight; for example osteoarthritis, heart disease, lipoma, hyperadrenocorticism, urinary incontinence, dystocia, cruciate disease and patellar luxation^12,24,25,27,28,40,52–58^. Confounders are defined as factors associated with both the risk factor and the outcome of interest but that are not on the causal pathway^21^. Age and bodyweight are therefore likely to act as confounders in analyses that aim to compare effects between brachycephalic and other skull shape groups but to date many studies have been reported using only univariable methods that fail to account for confounding and therefore potentially may report results that are heavily confounded and misleading.

The median age of any group of breeds is strongly influenced by whether the predominant breeds in the group are increasing or decreasing in popularity; increasing popularity will promote the introduction of many new puppies into the population and therefore shift the median age downwards with proportionately more younger dogs entering than there are older dogs dying^7^. The past decade has seen marked increases in popularity for several brachycephalic breeds in the UK, with proportional ownership of Chihuahua^59^, French Bulldog^18^ and Pug^20^ rising steeply. These rapid increases in popularity of these breeds contribute to a lowered median age of brachycephalic dogs overall. Although the median age of the brachycephalic group (3.31 years) was statistically lower than each of the other three groups, it is noteworthy that the median ages for crossbreds (3.74 years) was numerically much closer and younger than the median ages for mesocephalic (5.33 years) and dolichocephalic (5.07 years) types. The relative youth of the crossbred group may reflect the recent surge in popularity of designer crosses such as labradoodle and cockapoo that will have had the effect of pulling the median age of the overall crossbred group downwards^60^.

In addition to age and bodyweight differences, brachycephalic breeds were less likely to be neutered than the other three skull shape groups and also had some differential insurance status effects, suggesting that neutering and insurance should also be considered a priori as potential confounders. Associations between neutering have been reported for many disorders including urinary incontinence, cancer, joint disease and some behavioural consequences^61–68^. Proportional uptake of pet insurance is associated with the probability of diagnosis for several disorders including corneal ulceration, hyperadrenocorticism, cruciate disease, mast cell tumour, chronic kidney disease and patellar luxation^12,40,54,55,57,69^. Access to the financial support of pet insurance may reduce diagnostic constraints for both owners and veterinarians to promote greater clinical freedom and hence higher levels of diagnosis^70^.

This study revealed profound differences in inference when the same core data were analysed using either univariable or multivariable methods. Several findings were identified from multivariable methods that would have been missed if only univariable testing had been applied, including that brachycephalic dogs show increased odds of having at least one disorder compared to crossbreed types and two precise term disorders (otitis externa, skin cyst) that were at increased odds in brachycephalic breeds. Conversely, there were eight confounded findings that would have been accepted as significant if only univariable analysis had been applied but that did not show associations after accounting for confounding in multivariable analysis: periodontal disease, obesity, overgrown nail(s), retained deciduous tooth, lameness, skin mass, osteoarthritis and lipoma. Reporting false positive and false negative results in scientific studies carries increasingly detrimental risks for dog welfare as we move into the era of evidence based veterinary medicine and policy^70,71^. Results from canine health studies influence breed club health initiatives, research funding, animal charity campaigning and government policy^36,73–75^ and thus the reliability of research findings are critical if we are to optimise decision-making on future dog welfare strategies. Publishing results that are heavily confounded, especially where this form of bias is not explicitly acknowledged, also contributes to the current ‘reproducibility crisis’ in scientific reporting and promotes a more general distrust in scientific outputs and quality^76,77^.

The findings of the current study confirm that there are substantially different inferences gained from multivariable analysis compared with univariable analysis. This suggests that multivariable methods should be considered as the gold standard when analysing canine health data and that key confounding variables including bodyweight, age, sex, neuter and insurance status should be considered as standard default covariables in these analyses unless there is evidence to justify exclusion of these from analyses. Additionally, the results of previous breed predisposition studies that did not include multivariable methods should now be viewed with a more critical eye, given that these findings may harbour false positive and/or negative inferences that are challenging to now identify. This need for later re-analysis of previous studies also highlights the importance of depositing research data along with relevant confounding variables in open access repositories so that results can be retrospectively verified if authors choose to publish only univariable analyses of their data initially^78^.

In the current study, undesirable behaviour (precise level disorder) and behavioural disorders (group level disorder) showed decreased odds in brachycephalic breeds compared to non-brachycephalic breeds. Several factors may influence this finding, including both actual differences in the frequency of undesirable (and desirable) behaviours between breed types, but also differing perceptions and expectations by owners about what is normal, or desirable within these breeds. The current allure to ownership of brachycephalic breeds is partly based on perceived breed-associated positive behavioural factors, namely making good companion dogs, and being good breeds for households with children^79^. Owner expectations of what is ‘normal’ or ‘good’ behaviour for their breed is likely to influence the likelihood of veterinary presentation for perceived undesirable behaviour. In a recent study of brachycephalic ownership experiences, one-fifth of owners reported their dog behaved better than expected, and two-thirds met expectations, suggesting that the majority of brachycephalic dog owners appear satisfied with their dog’s behaviour^80^. Studies that explore actual behavioural differences (positive or negative traits) between brachycephalic and non-brachycephalic breeds are in their relative infancy compared to studies on physical health differences. However, initial findings suggest some potential divergences between these breed types, particularly in relation to dog–human communication and affiliation. Evidence suggests that brachycephalic dogs are more affectionate, cooperative and interactive with unfamiliar humans than dogs with relatively longer skulls^81,82^.

There are limitations to the current study. Breed status was assigned at the discretion of the owner and the veterinary team without validation based on pedigree records, so some breed misclassification was possible. The generalisability of the current results to countries outside of the UK and over time may vary. The median bodyweight of the brachycephalic and the non-brachycephalic groups will be heavily influenced by the dominant breeds within each group, which is liable to geographical variation, and demographic trends^83^. The distribution of breeds within the brachycephalic group in the current study is highly skewed towards a smaller number of very popular breeds in the UK, such as the Chihuahua, Shih Tzu, Cavalier King Charles Spaniel, French Bulldog and Pug, with these five breeds alone representing 71.87% of all dogs in this category. As such, more general characteristics of the overall brachycephalic group may be obscured by these breeds and biased towards small-medium brachycephalic dogs rather than less common, larger brachycephalic breeds such as the Dogue De Bordeaux and Bullmastiff. Future work specifically aimed at subsets of smaller and larger sized brachycephalic breeds can help to improve clarity based on body size. Research findings can be reported at differing levels of abstraction ranging from high abstraction (such as skull conformation) to moderate abstraction (such as breed) to precise abstraction (such as specific subsets of breeds)^84^. Research at different levels of abstraction offers differing advantages and drawbacks, and there is no single ideal abstraction level that answers research question. Research at very precise abstraction offers advantages of tighter application to well defined phenotypes of dogs (such as one specific disorder in one sex of one breed in one country) but conversely limits the proportion of overall dogs that are covered. Alternatively, higher levels of abstraction can assist our understanding of broader concepts such as skull conformation, but may be criticised for offering less rigour in relation to each of the many subtypes of dogs within these broad skull conformational groups. Consequently, although the current study applied a high level of abstraction to explore associations between general health and skull conformation, the cautious reader should not interpret this to infer that every breed or subtype of dog in each skull conformation category carries equal risk for these disorders. This study was based on the general population of dogs under primary-veterinary care in the UK but was unable to differentiate between Kennel Club registered and non-Kennel Club registered dogs which may differ in health status. The Kennel Club has recently made efforts to reduce points of concern for individual breeds, with brachycephalic breeds as a priority, using the Breed Watch scheme^85^ and is also working with the relevant breed clubs on defined breed health strategies within its Breed Health and Conservation Plans^73^. It is also noteworthy that this study explores effects associated with brachycephalism overall but the ecological fallacy phenomenon suggests that these effects may not necessarily apply to all breeds within these groups^86^. For example, brachycephalic dogs had 3.46 higher odds of heart murmur compared to non-brachycephalic types. However, over 10% of the brachycephalic group were Cavalier King Charles Spaniels, a breed highly predisposed to heart murmurs with a reported prevalence of 30.9%^37^. Although the results of the current study may assist to generate an overall view of the impact from brachycephalism on dog health, it is also clear that a breed-by-breed approach is additionally required to tackle specific problems that may differ in predisposition between breeds even within the brachycephalic group, for example corneal disorders in Pugs^20^ and skin fold pyoderma in Bulldogs^19^. Further than this, there is additionally wide variation in health status between individual dogs within each breed and thus ultimately each dog should be considered on its own individual merits for breeding, beyond the label of its breed. The grouping of dogs into skull categories, and the choice of categorisation scheme is another potential limitation of this work. Breeds were categorised by the authors based on typical breed-related skull-shape conformation but this process did not include measurements of individual skull conformation^11^ or apply format cut-points for category boundaries based on cephalic index^87^ or other skull metrices including craniofacial ratio^11^, craniofacial angle^88^, and skull index^89^. Supplementary A shows the breed categorisation that was used in the study. There is currently no standardised classification system that comprehensively links the spectrum of dog breeds to skull shape. The classification (Appendix A) used in the current study is the result of work by the authors over the past several years but is still open to update based on new information and opinions. Although brachycephalic, mesocephalic and dolichocephalic are useful classifiers to capture the wide variety in dog skull shapes, they have been criticised as overly simplistic and likely to miss subtle differences in head shape within each category^90^. Indeed, recent studies have identified more subtle elements of skull conformation that are risk factors for disorders such as syringomyelia^91^. Although potentially hampered by these limitations, the methods used in the current study are bolstered by the application of the big data approach, which has the power to identify differences between these groups and generate hypotheses for further, more in-depth studies. The results of the many specific statistical comparisons reported in the current study should be taken as exploratory rather than confirmatory; the authors were aiming to explore general principles of comparison between the skull shape categories and between univariable versus multivariable methods rather than to confirm predispositions for the specific disorders.

## Conclusion

This study provides strong evidence to support the common assertion that brachycephalic breeds are generally less healthy than their non-brachycephalic counterparts in relation to total disorder counts and specific common conditions recorded. Potential solutions to some of these health problems are likely to require conformational change to current skull shapes averages for many breeds; however, many other health problems will require targeted action at the individual breed level, owing to large differences in individual breed predispositions to disorders. Results from studies that report only univariable methods should be treated with extreme caution due to potential confounding effects that have not been accounted for during study design or analysis.

## Methods

The study population included all available dogs under primary veterinary care at clinics distributed across the entire of the UK that were participating in the VetCompass Programme during 2016. Dogs under veterinary care were defined as those with either a) at least one electronic patient record (EPR) (free-text clinical note, treatment or bodyweight) recorded during 2016 or b) at least one EPR recorded during both 2015 and 2017. VetCompass collates de-identified EPR data from primary-care veterinary practices in the UK for epidemiological research^30^. Data fields available to VetCompass researchers include a unique animal identifier along with species, breed, date of birth, sex, neuter status, insurance status and bodyweight, and clinical information from free-form text clinical notes and treatment with relevant dates.

A cross-sectional analysis using cohort clinical data was used to estimate the one-year (2016) period prevalence of the most commonly diagnosed disorders in brachycephalic, mesocephalic and dolichocephalic dog types^92^. Sample size calculations estimated that approximately 3,346 brachycephalic types and 13,384 non-brachycephalic types would be needed to detect an odds ratio ≥ 1.50 for any disorder with ≥ 1.50% prevalence in the non-brachycephalic group, assuming 1:4 ratio of brachycephalic to non-brachycephalic dog types (80% power and 95% confidence)^93^. Ethics approval was obtained from the RVC Ethics and Welfare Committee (Reference SR2018-1652). All methods were performed in accordance with the relevant guidelines and regulations. Informed consent for use of the clinical data of the study dogs was obtained from all of the participating clinics and the animal owners.

Breed status was assigned by the participating practices based on information provided by the owners in combination with the opinion of the veterinary professional teams. The recorded breed status could be updated over time in the clinical records. The latest available breed status was used in the current study, based on the assumption that accuracy would improve over time. Breed status was cleaned and mapped to a VetCompass breed list derived and extended from the VeNom Coding breed list^94^. Breeds were categorised by the authors into four groups based on typical skull-shape conformation^41^: brachycephalic, mesocephalic, dolichocephalic and crossbred dog types (Supplementary A). Crossbred dogs included all dogs that were not recorded with a standard recognised breed name^41, 95^; crossbreds included genuine ‘mixed-breed’ mongrels as well as dogs where some breed parentage information was recorded including those that are so-called designer types such as labradoodle and cockapoo^60^. Mesocephalic, dolichocephalic and crossbred dog types were further grouped as non-brachycephalic types for the purposes of disease risk analyses. Neuter and insurance status were defined by the final available EPR value. Adult bodyweight was defined as the mean of all bodyweight (kg) values recorded for each dog after reaching 18 months old and was categorised: ≤ 5.0, 5.0 to < 10.0, 10.0 to < 15.0, 15.0 to < 20.0, 20.0 to < 25.0, 25.0 to < 30.0, 30.0 to < 35.0, 35.0 to < 40.0 and ≥ 40.0. Mean adult bodyweight was calculated for each breed/sex combination with adult bodyweight available for ≥ 100 dogs. Individual dogs were categorised as “at or above the breed/sex mean”, “below the breed/sex mean” and “no recorded bodyweight” compared with the relevant breed/sex category. Age (years) was defined at December 31, 2016 and categorised: ≤ 1.0, 1.0 to < 2.0, 2.0 to < 4.0, 4.0 to < 6.0, 6.0 to < 8.0, 8.0 to < 10.0, 10.0 to < 12.0 and ≥ 12.0.

The list of unique animal identification numbers was randomly ordered and the clinical records of a randomly selected subset of animals were reviewed in detail to extract the most definitive diagnoses recorded for all disorders with clinical evidence of existence during 2016^31^. For the current study, disorders were defined as conditions that show deviation from good health and are often characterised by functional impairment^96^. Elective (e.g. neutering) or prophylactic (e.g. vaccination) clinical events were not included. No distinction was made between pre-existing and incident disorders. Disorders described within the clinical notes using presenting sign terms (e.g. ‘vomiting’ or 'vomiting and diarrhoea'), but without a formally recorded clinical diagnostic term, were included using the first sign listed (e.g. vomiting). The extracted diagnosis terms were mapped to a dual hierarchy of diagnostic precision for analysis: precise terms and grouped terms as previously described^31^. Briefly, precise terms described the original extracted terms at the maximal diagnostic precision recorded within the clinical notes (e.g. *inflammatory bowel disease *would remain as *inflammatory bowel disease*). Grouped-level precision terms mapped the original diagnosis terms to a general level of diagnostic precision (e.g. *inflammatory bowel disease* would map to *gastro-intestinal*).

Following internal validity checking and data cleaning in Excel (Microsoft Office Excel 2013, Microsoft Corp.), analyses were conducted using Stata Version 13 (Stata Corporation). The sex, neuter status, insurance status, age, adult bodyweight and breed composition for common breeds were described and the one-year period prevalence values were reported with 95% confidence intervals (CI) that described the probability of diagnosis at least once during 2016 for brachycephalic, mesocephalic, dolichocephalic and crossbred dog types under veterinary care during 2016. The CI estimates were derived from standard errors based on approximation to the binomial distribution^97^.

Direct comparisons between variables other than disorders used the chi-square test to evaluate categorical variables (Fisher’s exact test was used if at least one of the reported cells was under 5) and the Mann–Whitney U test to evaluate binary categorical variables for association with continuous variables^97^. The odds of disorder occurrence were estimated using binary logistic regression. Univariable risk factor analyses directly compared the odds for each disorder between brachycephalic and non-brachycephalic dogs. Multivariable risk factor analyses applied mixed effects multivariable binary logistic regression modelling to evaluate associations between each disorder and the brachycephalic/non-brachycephalic factor of main interest along with a fixed set of covariables included to account for confounding (*adult bodyweight category, bodyweight relative to breed/sex mean, age category, sex, neuter* and *insurance*). Breed and clinic attended were included as a random effects^21^. Decision-making on which variables to include in these standard models used an ‘information theory’ approach to include a priori variables that the authors considered as potential confounders for outcome associations with the skull conformation variable that was of primary interest^98^. Multivariable Poisson regression modelling was used to evaluate associations between the skulls shape factor of main interest (brachycephalic, mesocephalic, dolichocephalic and crossbred) along with the same fixed set of covariables (*adult bodyweight category, bodyweight relative to breed/sex mean, age category, sex, neuter* and *insurance)* and the numerical outcome of the count of disorders recorded during 2016. Statistical significance was set at the 5% level. Only the results for the brachycephalic/non-brachycephalic factor of main interest are reported from each regression model.

### Ethics approval

Ethics approval was granted by the RVC Ethics and Welfare Committee (reference number SR2018-1652).

### Consent for publication

The Royal Veterinary College has provided permission to publish this paper. The manuscript number is PPH_02188.

## Supplementary information

Supplementary file1

## Data Availability

Original data used for the current study will be made freely available on the Royal Veterinary College Data Repository.

## Abbreviations

CI: Confidence interval
DCRR: Disorder count risk ratio
EPR: Electronic patient record
IQR: Interquartile range
KC: The Kennel Club
OR: Odds ratio
