# Supplementary material for: Unravelling the health status of brachycephalic dogs in the UK using multivariable analysis
Source: Sci Rep. 2020 Oct 14;10:17251. doi: 10.1038/s41598-020-73088-y (PMC7560694; doi:10.1038/s41598-020-73088-y)
Supplement: Supplementary file 1 — Supplementary file1 [file 41598_2020_73088_MOESM1_ESM.docx]

**Unravelling the health status of brachycephalic dogs in the UK using multivariable analysis**

O’Neill, DG^1^; Pegram, C^1^; Crocker, P^1^; Brodbelt, DC^1^; Church, DB^2^; Packer, RMA^2^

^1^Pathobiology and Population Sciences, The Royal Veterinary College, Hawkshead Lane, North Mymms, Hatfield, Herts AL9 7TA, UK

^2^Clinical Sciences and Services, The Royal Veterinary College, Hawkshead Lane, North Mymms, Hatfield, Herts AL9 7TA, UK

*Corresponding author: Dan G. O’Neill *, MVB BSc(hons) MSc(VetEpi) PhD FRCVS, Pathobiology and Population Science, The Royal Veterinary College, Hawkshead Lane, North Mymms, Hatfield, Herts AL9 7TA, UK [doneill@rvc.ac.uk](mailto:doneill@rvc.ac.uk)

Supplementary A: Individual breeds grouped as brachycephalic (n = 34), mesocephalic (n = 169) and dolichocephalic (n = 66) among dogs under UK primary veterinary care at practices participating in the VetCompass Programme.

| Brachycephalic | Mesocephalic | Dolicocephalic |
| --- | --- | --- |
| Affenpinscher | African Sand Dog | Airedale Terrier |
| Alapaha Blue Blood Bulldog | Alaskan Klee Kai | American Bull Terrier |
| American Bandogge Mastiff | Alaskan Malamute | Andalusian Mouse-Hunting Dog |
| American Bulldog | American Akita | Australian Kelpie |
| Boston Terrier | American Cocker Spaniel | Basset Griffon Vendeen |
| Boxer | American Foxhound | Basset Hound |
| British Bulldog | American Hairless Terrier | Bedlington Terrier |
| Bull Boxer | American Mastiff | Belgian Malinois Shepherd Dog |
| Bull Mastiff | American Pit Bull Terrier | Belgian Shepherd |
| Bulldog | American Staffordshire Terrier | Bleu de Gascogne Basset |
| Cavalier King Charles Spaniel | Anatolian Karabash Shepherd Dog | Bloodhound |
| Chihuahua | ASCOB Cocker Spaniel | Borzoi |
| Dogue de Bordeaux | Australian Cattle Dog | Bracco Italiano |
| Dorset Olde Tyme Bulldogge | Australian Sheepdog | Bull Terrier |
| English Mastiff | Australian Shepherd Dog | Carpathian Sheepdog |
| French Bulldog | Australian Silky Terrier | Croatian Sheepdog |
| German Boxer | Australian Terrier | Dachshund |
| Griffon | Basenji | English Bull Terrier |
| Griffon Bruxellois | Beagle | English Greyhound |
| Italian Mastiff | Bearded Collie | Fox Terrier |
| Japanese Chin | Bergamasco Shepherd Dog | German Pinscher |
| King Charles Spaniel | Bernese Mountain Dog | German Shepherd Dog |
| Lhasa Apso | Bichon Frise | German Shepherd Dog - White |
| Long-Haired Chihuahua | Biewer Terrier | Grand Griffon Vendeen |
| Mastiff | Black Russian Terrier | Great Dane |
| Miniture Shih-Tzu | Bolognese Bichon | Greyhound |
| Neopolitan Mastiff | Border Collie | Ibizan Hound |
| Olde English Bulldogge | Border Terrier | Irish Terrier |
| Pekingese | Bouvier des Flandres | Irish Wolfhound |
| Pug | Briard | Italian Greyhound |
| Shih-tzu | Brittany Spaniel | Kerry Blue Terrier |
| Short-Haired Chihuahua | Cairn Terrier | Lakeland Terrier |
| Teacup Chihuahua | Cardigan Welsh Corgi | Manchester Terrier |
| Victorian Bulldog | Catalan Sheepdog | Miniature Bull Terrier |
|  | Caucasian Sheepdog | Miniature Dachshund |
|  | Central Asian Sheepdog | Miniature Doberman Pinscher |
|  | Chinese Crested | Miniature English Bull Terrier |
|  | Chinese Shar-Pei | Miniature Long-Haired Dachshund |
|  | Chow Chow | Miniature Pinscher |
|  | Cocker Spaniel | Miniature Poodle |
|  | Collie | Miniature Smooth-Haired Dachshund |
|  | Coton De Tulear | Miniature Wire-Haired Dachshund |
|  | Dalmatian | Petit Basset Griffon Vendeen |
|  | Dandie Dinmont Terrier | Pinscher |
|  | English Cocker Spaniel | Poodle |
|  | English Pointer | Saluki |
|  | English Setter | Scottish Deerhound |
|  | English Springer Spaniel | Scottish Rough Collie |
|  | English Toy Terrier | Scottish Smooth Collie |
|  | Estrela Mountain Dog | Scottish Terrier |
|  | Eurasier | Shetland Sheepdog |
|  | Fauve de Bretagne Basset | Small Portuguese Podengo |
|  | Fell Terrier | Smooth Fox Terrier |
|  | Field Spaniel | Standard Dachshund |
|  | Finnish Lapphund | Standard Doberman Pinscher |
|  | Finnish Spitz | Standard Long-Haired Dachshund |
|  | Flat Coated Retriever | Standard Poodle |
|  | German Long-Haired Pointer | Standard Smooth-Haired Dachshund |
|  | German Pointer | Standard Wire-Haired Dachshund |
|  | German Short-Haired Pointer | Toy Fox Terrier |
|  | German Spitz | Toy Poodle |
|  | German Wire-Haired Pointer | Transylvanian Hound |
|  | Giant Schnauzer | Welsh Terrier |
|  | Glen of Imaal Terrier | Whippet |
|  | Golden Retriever | Wire-Haired Fox Terrier |
|  | Gordon Setter | Wolfhound |
|  | Greek Hound |  |
|  | Greek Shepherd |  |
|  | Hairless Chinese Crested |  |
|  | Harrier Beagle |  |
|  | Havanese |  |
|  | Hound |  |
|  | Hungarian Kuvasz |  |
|  | Hungarian Vizsla |  |
|  | Hungarian Water Dog |  |
|  | Husky |  |
|  | Inuit Dog |  |
|  | Irish Red and White Setter |  |
|  | Irish Red Setter |  |
|  | Irish Staffordshire Bull Terrier |  |
|  | Irish Water Spaniel |  |
|  | Italian Spinone |  |
|  | Jack Russell Terrier |  |
|  | Japanese Inu Akita |  |
|  | Japanese Shiba Inu |  |
|  | Japanese Spitz |  |
|  | Kai Dog |  |
|  | Keeshond |  |
|  | Kromfohrländer |  |
|  | Kyi-Leo |  |
|  | Labrador Husky |  |
|  | Labrador Retriever |  |
|  | Lagotto Romagnolo |  |
|  | Lancashire Heeler |  |
|  | Leonberger |  |
|  | Leopard Cur |  |
|  | Lowchen |  |
|  | Lucas Terrier |  |
|  | Maltese |  |
|  | Miniature Jack Russell Terrier |  |
|  | Miniature Schnauzer |  |
|  | Miniature Yorkshire Terrier |  |
|  | Mountain Cur |  |
|  | Munsterlander Large Pointer |  |
|  | Newfoundland |  |
|  | Norfolk Terrier |  |
|  | North American Shepherd Dog |  |
|  | Northern Inuit Dog |  |
|  | Norwegian Elkhound |  |
|  | Norwegian Sheepdog |  |
|  | Norwich Terrier |  |
|  | Nova Scotia Duck Tolling Retriever |  |
|  | Old English Sheepdog |  |
|  | Ovtcharka |  |
|  | Papillon |  |
|  | Parson Russell Terrier |  |
|  | Patterdale Terrier |  |
|  | Pembroke Welsh Corgi |  |
|  | Perro de Presa Canario |  |
|  | Plummer Terrier |  |
|  | Pointer |  |
|  | Polish Lowland Sheepdog |  |
|  | Pomeranian |  |
|  | Portuguese Water Dog |  |
|  | Powder Puff Chinese Crested |  |
|  | Prague Ratter |  |
|  | Pyrenean Mountain Dog |  |
|  | Pyrenean Shepherd Dog |  |
|  | Rat Terrier |  |
|  | Retriever |  |
|  | Rhodesian Ridgeback |  |
|  | Roman Ridgeback |  |
|  | Rottweiler |  |
|  | Russian Toy Terrier |  |
|  | Saint Bernard |  |
|  | Samoyed |  |
|  | Schnauzer |  |
|  | Sealyham Terrier |  |
|  | Setter |  |
|  | Sheepdog |  |
|  | Siberian Husky |  |
|  | Skye Terrier |  |
|  | Soft-Coated Wheaten Terrier |  |
|  | Spaniel |  |
|  | Spanish Pointer |  |
|  | Spanish Water Dog |  |
|  | Spitz |  |
|  | Springer Spaniel - Unspecified |  |
|  | Staffordshire Bull Terrier |  |
|  | Standard Alaskan Husky |  |
|  | Standard Schnauzer |  |
|  | Sussex Spaniel |  |
|  | Tahltan Bear Dog |  |
|  | Tamaskan Husky |  |
|  | Terrier |  |
|  | Tibetan Mastiff |  |
|  | Tibetan Spaniel |  |
|  | Tibetan Terrier |  |
|  | Toy German Spitz |  |
|  | Toy Terrier |  |
|  | Weimaraner |  |
|  | Welsh Corgi |  |
|  | Welsh Sheepdog |  |
|  | Welsh Springer Spaniel |  |
|  | West Highland White Terrier |  |
|  | Wire-Haired Hungarian Vizsla |  |
|  | Wirehaired Jack Russell Terrier |  |
|  | Working Cocker Spaniel |  |
|  | Yorkshire Terrier |  |
